# Evolution of Structural Diversity of Triterpenoids

**DOI:** 10.3389/fpls.2019.01523

**Published:** 2019-12-17

**Authors:** Pablo D. Cárdenas, Aldo Almeida, Søren Bak

**Affiliations:** Department of Plant and Environmental Science, University of Copenhagen, Frederiksberg, Denmark

**Keywords:** triterpenoid saponins, structural diversity, convergent evolution, plant specialized metabolism, unlinked versus clustered pathways

## Abstract

Plants have evolved to produce a blend of specialized metabolites that serve functional roles in plant adaptation. Among them, triterpenoids are one of the largest subclasses of such specialized metabolites, with more than 14,000 known structures. They play a role in plant defense and development and have potential applications within food and pharma. Triterpenoids are cyclized from oxidized squalene precursors by oxidosqualene cyclases, creating more than 100 different cyclical triterpene scaffolds. This limited number of scaffolds is the first step towards creating the vast structural diversity of triterpenoids followed by extensive diversification, in particular, by oxygenation and glycosylation. Gene duplication, divergence, and selection are major forces that drive triterpenoid structural diversification. The triterpenoid biosynthetic genes can be organized in non-homologous gene clusters, such as in *Avena* spp., Cucurbitaceae and *Solanum* spp., or scattered along plant chromosomes as in *Barbarea vulgaris*. Paralogous genes organized as tandem repeats reflect the extended gene duplication activities in the evolutionary history of the triterpenoid saponin pathways, as seen in *B. vulgaris*. We review and discuss examples of convergent and divergent evolution in triterpenoid biosynthesis, and the apparent mechanisms occurring in plants that drive their increasing structural diversity within and across species. Using *B. vulgaris*’ saponins as examples, we discuss the impact a single structural modification can have on the structure of a triterpenoid and how this affect its biological properties. These examples provide insight into how plants continuously evolve their specialized metabolome, opening the way to study uncharacterized triterpenoid biosynthetic pathways.

## Introduction

Plants have evolved the strategy of constantly diversifying the chemical structures they produce, evolving into an astonishing number of so-called plant specialized metabolites—of which the majority are thought to be involved in plant defense. The general conception has been that divergent evolutionary processes is the major driving force. However, biochemical and molecular knowledge of how these compounds have evolved has demonstrated that convergent evolution is surprisingly common (Pichersky and Lewinsohn, 2011).

**Figure 1:**
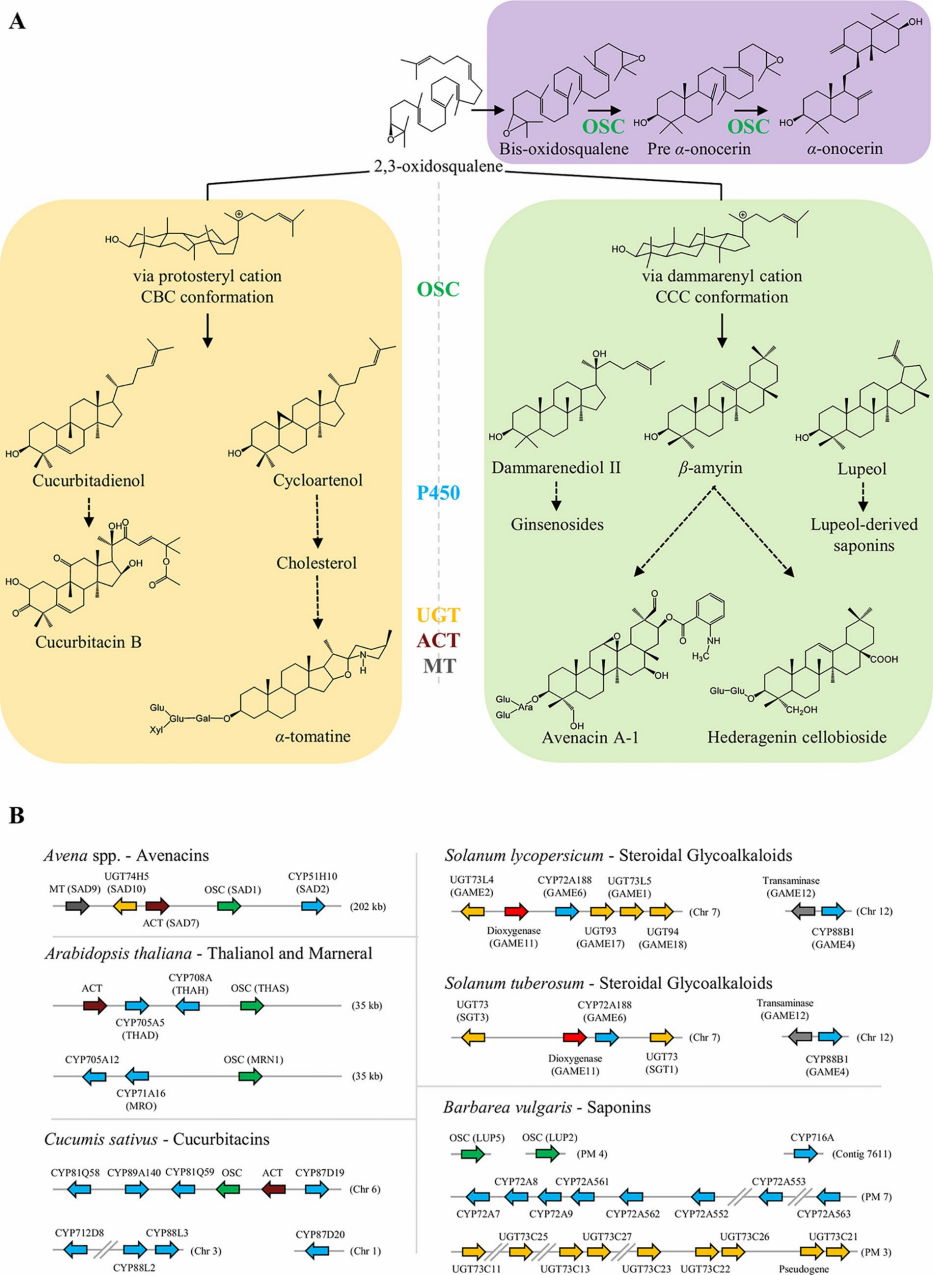
Simplified representation of the biosynthesis of sterols and triterpenoids in plants. **(A)** OSC signature enzymes catalyze the cyclization of 2,3-oxidosqualene, and in more rare cases bis-oxidosqualene, into several triterpenoid scaffolds. These structures can be further modified by tailoring enzymes, including oxygenation by P450s, glycosylation by UGTs, acylation by ACT, and methylation by MT. Selected structures are depicted and discussed in more detail in the text. Dashed arrows represent multiple biosynthetic reactions whereas solid arrows represent a single step. **(B)** Biosynthesis of plant triterpenoids can be mediated by non-homologous clustered genes or through non-linked genes. In *Avena* spp., a cluster of five genes are involved in the biosynthesis of avenacin A-1. In *Arabidopsis thaliana*, two clusters have been reported: thalianol cluster with four genes (up) and marneral cluster with three genes (down). In *Cucumis sativus*, six genes associated with cucurbitacin biosynthesis are located in a cluster in chromosome 6, while four other genes are elsewhere in the genome. The core genes for biosynthesis of SGAs are clustered in chromosome 7 and 12 of *S. tuberosum* and *S. lycopersicum*, respectively. The key genes for biosynthesis of the insect-feeding deterrent hederagenin cellobioside are distributed along *B. vulgaris* genome in tandem repeats located at different pseudomolecules (PM). OSC, oxidosqualene cyclase; P450, cytochrome P450; UGT, UDP-glycosyltransferase; ACT, acyltransferase; MT, methyltransferase.

The increasing availability of genome sequencing of evolutionary distant plant species and in-depth studies of triterpenoid pathways in recent years, has revealed an emerging picture that triterpenoid pathways are evolving recurrently and thus represent an interesting case of both divergent and convergent evolution. Typically, it is the same gene families (OSCs, P450s, and UGTs) that are constantly recruited and afterwards expanded through gene duplications leading to tandem repeats. Here, we review the current knowledge about five triterpenoid scaffolds (and their derivatives) as examples of both divergent and convergent evolution in specialized metabolism and discuss the selection pressure on the genes, their organization, and how plants cope with the toxic triterpenoids they produce.

## Convergent Evolution in Biosynthesis of Triterpenoid Scaffolds

### Dammarenyl Cation-Derived Triterpenoids Have Arisen Multiple Times During Angiosperm Evolution

The majority of the known triterpenoids arise from the dammarenyl cation (Xiong et al., 2005). Resins from trees of the Dipterocarpaceae are known as dammar, hence the first triterpenoids isolated from dammar resins were coined dammaranediol I and II (Mills and Werner, 1955; Mills, 1956). Currently, the most commercially-valuable representatives of this class of compounds are the diverse ginsenosides. They can be obtained from mature six-year-old rhizomes of *Panax ginseng* and because of their anticancer activities some ginsenosides are used for chemotherapy treatment (Leung and Wong, 2010). Tansakul et al. (2006) were the first to clone a Dammarenediol-II synthase (PgDDS) and showed that this OSC from *P. ginseng* catalyzes the first committed step of ginsenoside biosynthesis. The only other DDS characterized thereafter belongs to *Centella asiatica* (Kim et al., 2009). *P. ginseng* and *C. asiatica* belong to the Apiales and phylogenetic analysis showed that both DDS grouped in the same branch suggesting the DDS in these species evolved from a common ancestor. As DDSs have not been elucidated from the phylogenetically distant Dipterocarpaceae, future work on this family will shed some light on the evolutionary history of DDSs and could indicate if they arose from convergent or divergent evolution.

Lupeol and β-amyrin are prevalent pentacyclic triterpenoids derived from the dammarenyl cation and they are ubiquitously found in many different plant species (**Figure 1**). Nevertheless, phylogenetic analysis have shown that the genes producing these scaffolds group distinctively in different clades. Shibuya et al. (1999) first distinguished two clades of lupeol synthases in plants; one which is composed of specific lupeol synthases and another which is composed of multi-functional OSCs producing α-, β-amyrin, and lupeol (Thimmappa et al., 2014; Khakimov et al., 2015). Site-directed mutagenesis experiments have shown that a single amino acid replacement could convert a lupeol synthase into a β-amyrin synthase (and conversely), indicating the noticeable role of specific residues may have played in the evolution of OSC product specificity and generation of triterpenoid diversity (Kushiro et al., 1999; Kushiro et al., 2000). Furthermore, phylogenetic analysis of both monocot and dicot OSCs by Xue et al. (2012) and Augustin et al. (2011), additionally distinguish two distinctive clades of β-amyrin synthase in monocots and dicots.

Lupeol and β-amyrin can be present in plants as unmodified compounds typically found in resins or waxes (Szakiel et al., 2012) or they have a major role as precursors for other specialized triterpenoid metabolites, usually involved in plant defense and development. Lupeol is involved in nodule formation in *Lotus japonicus* through regulation of *ENOD40* gene expression (Delis et al., 2011). Lupeol is also part of the cuticular wax surface of castor bean plant (*Ricinus communis*) where it was suggested to have a physiological role in protection against dehydration (Guhling et al., 2006). Additionally, lupeol is the precursor for betulinic acid, a triterpenoid that accumulates in the bark of birch tree and has a potent anticancer activity (Pisha et al., 1995; Kumar et al., 2018). β-amyrin is a precursor of glycyrrhizin, a triterpenoid saponin found in the roots and stolons of liquorice (*Glycyrrhiza glabra*) well known for its pharmaceutical properties and as a natural sweetener (Hayashi et al., 2002). β-amyrin is also precursor for the antifungal saponin avenacin A-1 in oat and for the potent insect-feeding deterrent hederagenin glycosides in the Brassicaceae *Barbarea vulgaris* (Kuzina et al., 2009; Nielsen et al., 2010; Khakimov et al., 2016; Liu et al., 2019);. Additionally, β-amyrin seems to play a role in root development in oat (Kemen et al., 2014) and in *Lotus japonicus* (Krokida et al., 2013), suggesting that triterpenoids like lupeol and β-amyrin are not exclusively involved in plant defense.

### α-Onocerin—A *Seco*-Triterpenoid (*sensu lato*) That Evolved Convergently

*Seco*-triterpenoids are characterized by the absence of a C-C bond that would normally form one of the consecutive rings in a triterpenoid scaffold. They are known to be distributed across the plant kingdom. The *seco*-triterpenoid (*sensu lato*) α-onocerin consists of two bicyclic systems connected by a two-carbon linkage; its occurrence appears to be limited to Lycopod species and some species within the *Ononis* genus in the Fabaceae. Lycopods and the Fabaceae originated in very distant evolutionary times (Garratt et al., 1984; Giraud and Lejal-Nicol, 1989), which implies that the α-onocerin trait evolved convergently in Lycopods and in the *Ononis* genus. The biological function of α-onocerin still remains unknown.

On a biochemical level, α-onocerin biosynthesis differs from other triterpenoids as it is biosynthesized from 2,3;22,23-oxidosqualene (bis-oxidosqualene) instead of the typical triterpenoid precursor 2,3-oxidosqualene (**Figure 1**). In *Lycopodium clavatum*, biosynthesis of α-onocerin is carried out in two sequential steps by two paralogous OSCs (Araki et al., 2016). Pre-α-onocerin synthase (LcLCC), initiates cyclization from one of the epoxide bonds of bis-oxidosqualene. The cyclization terminates after formation of the A and B rings through the generation of pre-α-onocerin. Subsequently α-onocerin synthase (LcLCD) carries out the cyclization of the D and E rings from the remaining epoxide ring, to yield α-onocerin. Our group demonstrated that *Ononis spinosa *α-onocerin is biosynthesized by a single OSC (OsONS1) (Almeida et al., 2018). In *O. spinosa* a neofunctionalized squalene epoxidases (OsSQEs) provide the OSCs with the necessary bis-oxidosqualene. Fluorescence imaging microscopy experiments demonstrated protein-protein interactions between OsONS1 and the neofunctionalized OsSQEs (Almeida et al., 2018).

Phylogenetic analysis revealed that OsONS1 branches off from lupeol synthases, and is phylogenetically distant from the two *L. clavatum *α-onocerin synthases which branch off directly from sterol biosynthesis; thus, α-onocerin biosynthesis evolved convergently in these two plant species (Almeida et al., 2018). In addition, molecular docking simulation showed that OsONS1 alone produces α-onocerin in two cyclization steps, as opposed to the specialized LcLCC and LcLCD in Lycopods.

### Protosteryl Cation-Derived Triterpenoids Are Found Across Angiosperm Phylogeny

Cucurbitacins are highly oxygenated tetracyclic triterpenes initially discovered in members of the Cucurbitaceae family and well known for their bitterness and toxicity (Metcalf et al., 1980). Cucurbitacins have been reported in 17 taxonomically distant related families in eudicots and some monocots. They consist of hundreds of derivatives from 20 main cucurbitacin molecules named from cucurbitacin A to T (Chen et al., 2005). At least 100 species in 30 genera of the Cucurbitaceae have been shown to contain cucurbitacins (Raemisch and Turpin, 1984), with cucurbitacin B (**Figure 1A**) being present in 91% of the species (Chen et al., 2005).

Cucurbitacins are extremely toxic to mammals (David and Vallance, 1955) and function as feeding deterrents against several insects (Nielsen et al., 1977; Bigger and Chaney, 1998) but they have also been found to be feeding stimulant for leaf beetles belonging to the Luperini tribe of the Chrysomelidae (Metcalf, 1986). Cucurbitacin B can displace the insect steroidal hormone 20-hydroxyecdysone, thus affecting morphological changes in *Drosophila melanogaster* (Dinan et al., 1997).

The cucurbitacin biosynthesis pathway has mainly been studied in members of the Cucurbitaceae. The OSC cucurbitadienol synthase from *Cucurbita pepo* (CpCPQ) cyclizes 2,3-oxidosqualene to cucurbitadienol (**Figure 1A**) (Shibuya et al., 2004). The CpCPQ gene evolved after the divergence of dicots and monocots when the ancestral cycloartenol synthase gene duplicated, creating two clades of cycloartenol synthases (CASI and CASII) of which CpCPQ evolved from the *C. pepo* cycloartenol synthase (CpCPX) in the CASII clade (Xue et al., 2012).

Cucurbitacins are also present outside the Cucurbitaceae; for example *Iberis amara* belonging to the Brassicaceae contain cucurbitacins (Nielsen et al., 1977). The elucidation of the cucurbitacin biosynthetic pathway in *Iberis* will help to clarify the evolutionary history of Cucurbitacin biosynthesis.

Steroidal glycoalkaloids (SGAs) are triterpene-derived compounds found in major dicot Solanaceae crops such as tomato and potato. SGAs contain a nitrogen incorporated on their steroidal scaffold and they provide the plant with a barrier against a broad range of herbivores and pathogens, but are bitter and considered anti-nutritional compounds for humans (Cárdenas et al., 2015). SGAs and non-nitrogenous steroidal saponins are also present in distantly related monocot Liliaceae plant species. In these plant species, they have had special attention due to their pharmacological properties. For instance, the Liliaceae *Veratrun californicum* produces the potent anticancer molecule cyclopamine (Augustin et al., 2015). Biochemical and phylogenetic analysis of the enzymes involved in the biosynthesis of *Solanum* and *Veratrum* SGAs suggested their convergent origins and their partial recruitment from primary phytosterol metabolism (Augustin et al., 2015; Sonawane et al., 2016).

## Genome Organization of Triterpenoid Pathways

The biosynthetic genes for triterpenoids display different genome organizations across plant species ((**Figure 1B**). While in some species they are arranged in clusters of non-homologous genes (e.g., avenacins in oat, Qi et al., 2004; cucurbitacins in cucumber, Shang et al., 2014); the biosynthesis of other triterpenoids is mediated by genes scattered along the plant genome organized typically in tandem repeats (e.g., saponins in *Barbarea vulgaris*, Khakimov et al., 2015; Erthmann et al., 2018; Liu et al., 2019; and mogrosides in Siraitia grosvenorii, Itkin et al., 2016).

### Clustered Genes Mediating Biosynthesis of Avenacins, Cucurbitacins and Steroidal Glycoalkaloids

Organization of genes in operon-like clusters has typically been associated with fungal genomes and operons present in bacteria. Nevertheless, nearly 20 metabolic gene clusters for plant specialized metabolites have been reported in multiple plant species (Boycheva et al., 2014). The avenacin, thalianol, and marneral triterpenoid biosynthetic pathways were among the first to be reported being organized in non-homologous gene clusters in oat and *Arabidopsis thaliana*, respectively ((**Figure 1B**, Qi et al., 2004; Field and Osbourn, 2008; Field et al., 2011) The organization of metabolic genes in clusters is thought to provide both co-inheritance, co-regulation and avoiding accumulation of toxic intermediates and may thus be beneficial for securing stable inheritance of functional chemical defense pathways in a dynamic ecological context of natural populations (Osbourn, 2010; Takos and Rook, 2012).

Triterpenoid saponins are found mainly in dicotyledonous species. *Avena* spp. is the only known triterpenoid saponin producing monocotyledon. *Avena* spp. accumulates avenacins which are saponins produced in the roots of oat species and provide the plant with a potent barrier against soil-borne fungi (Papadopoulou et al., 2002) (**Figure 1**). SAD1, a β-amyrin synthase, catalyzes the first committed step in avenacin biosynthesis (Qi et al., 2004), and is present in the oat genome in an operon like cluster together with genes coding for the tailoring enzymes required for avenacin biosynthesis, including: SAD2, the P450 CYP51H10 that oxidizes β-amyrin; and three genes that act together in the acylation steps of avenacin: SAD9, a N-methyltransferase; SAD10, a UGT74H5 glycosyltransferase; and SAD7, a serine carboxypeptidase-like acyltransferase (Mugford et al., 2013). CYP51H10 (SAD2) evolved from a predisposition in sterol metabolism of the orthologous CYP51 catalyzing the conserved 14α-demethylation reaction in sterol metabolism in eukaryotes (Geisler et al., 2013).

In Cucurbitaceae, part of the cucurbitacin pathway is similarly clustered; in cucumber the cucurbitadienol synthase is flanked by four P450s and an ACT gene out of which two P450s and the ACT have been functionally characterized (Shang et al., 2014). The P450s in this cluster belong to different subfamilies, indicating this cluster was not formed by tandem duplications but by genome reorganization. Later Zhou et al. (2016) uncovered the same conserved syntenic loci in melon and watermelon by comparative analyses of their genomes. While the core cluster is overall syntenically conserved, the CYP88A60 catalyzing the hydroxylation at position C19 and the CYP87D20 catalyzing hydroxylations at C11 and C20 both lie outside of the non-homologous gene cluster.

In Solanaceae, the core genes required for the biosynthesis of steroidal glycoalkaloids are part of a metabolic operon-like gene cluster (**Figure 1B**). Itkin et al. (2013) showed that in tomato, six SGA genes are arranged in a cluster on chromosome 7 (UGT73L5: GAME1; UGT93: GAME17; UGT94: GAME18; UGT73L4: GAME2; Dioxygenase: GAME11; and CYP72A188: GAME6), whereas two other genes are located next to each other on chromosome 12 (CYP88B1: GAME4; and Transaminase: GAME12). Similarly in potato, four SGA-related genes are located in chromosome 7 (UGT73: SGT3; Dioxygenase: GAME11; CYP72A188: GAME6; and UGT73: SGT1) and two in chromosome 12 (Transaminase: GAME12; and CYP88B1: GAME4).

### Tandem Repeats of Triterpenoid-Biosynthetic Genes and Triterpenoid Diversity in *B. vulgaris*, *Medicago truncatula* and *Glycyrrhiza uralensis*

Triterpenoid saponin biosynthesis has evolved recurrently in evolution and thus the organization of the genes may not be conserved. In recent years, the *Barbarea* genus has appeared as a unique plant model as it is the only genus in the agronomically important cabbage family (Brassicaceae) known to accumulate saponins (Khakimov et al., 2016). Some of these saponins (e.g., hederagenin cellobioside) are highly deterrent to *Brassica* specialist herbivores such as flea beetles (*Phyllotreta nemorum*) and the diamondback moth (*Plutella xylostella*) (Kuzina et al., 2009; Kuzina et al., 2011; Liu et al., 2019).

Genome (Byrne et al., 2017) and genetic (Kuzina et al., 2009; Kuzina et al., 2011) analysis showed that the genes mediating the biosynthesis of the deterrent triterpenoid saponins in *B. vulgaris* are not linked, but are present in tandem repeats spread along the genome (**Figure 1B**). The OSCs, P450s, and UGTs in the pathway are generally characterized to be rather substrate and product promiscuous enzymes which may facilitate that more than 49 different saponin structures can be generated in *B. vulgaris* with a limited number of genes (Khakimov et al., 2016). QTLs for flea beetle resistance and accumulation of saponins co-localize in *B. vulgaris* (Kuzina et al., 2009; Kuzina et al., 2011; Khakimov et al., 2015). Two unlinked QTLs containing two OSC (i.e., LUP2 and LUP5) and a tandem repeat of eight P450s (CYP72As), respectively, were identified. *In vitro* and *in planta* assays shown that LUP5 was preferentially expressed in the insect-deterrent *B. vulgaris* G-type. LUP5 leads to predominant accumulation of β-amyrin, the precursor for the deterrent hederagenin cellobioside. Conversely, LUP2 was preferentially expressed in the P-type plants (insect-susceptible), and produces mainly lupeol, the precursor for lupeol-derived saponins that appear not to be deterrent. Of the eight CYP72As, only CYP72A552 oxidizes oleanolic acid at the C23 position leading to the formation of insect deterrent hederagenin glycosides (Liu et al., 2019). CYP716A80 and CYP716A81 catalyze C28 carboxylation and two additional hydroxylation (Khakimov et al., 2015) while a tandem repeat of at least five UGT73Cs (Augustin et al., 2012; Erthmann et al., 2018) are also involved in the pathway. Both the CYP716As and the UGT73Cs are not linked to QTLs for neither insect resistance nor saponin accumulation. All members of the UGT73C tandem repeat accepted both hederagenin and oleanolic acid as substrates, but generated different glycosylated products, creating an spectrum of mono and bisdesmosidic saponins (Erthmann et al., 2018). If the *B. vulgaris* saponin non-linked gene organization is part of facilitating the opportunity to create multiple end products with a number of limited genes is an open question, but interestingly other pathways (e.g., anthocyanins and glucosinolates) also producing a multiple range of end products, are also known to be unlinked.

As in *B. vulgaris*, the genome of *Medicago truncatula* and the draft genome of *Glycyrrhiza uralensis* did not reveal operon-like gene cluster of OSCs, P450s, and UGTs involved in neither soyasaponins nor glycyrrhizin biosynthesis, but rather the gene candidates are organized as tandem repeats of each of these gene families (Naoumkina et al., 2010; Mochida et al., 2017). However, while most of the genes in these tandem repeats remain functionally uncharacterized, UGT73K1 was proven to glycosylate soyasapogenol B and E at the C3 position (Achnine et al., 2005).

## What Determines Toxicity Versus Autotoxicity of Plant Triterpenoid Saponins?

Saponins are generally assumed to target and disrupt vital functions common among organisms, such as the integrity of the cell membrane. In this regard, a fundamental paradox in plant evolution is that many defense metabolites may be harmful for the plant themselves. Therefore, plants have had to evolve strategies to both biosynthesize and accumulate defense molecules without causing autotoxicity. Some of the strategies used by plants to avoid autotoxicity are the compartmentalization of defense compounds in specialized structures and chemical modifications leading to decreased toxicity (i.e., glycosylation). For instance, avenacin A-1 is normally localized in the vacuoles of root epidermal cells. Mutants for the *sad3* glucosyltransferase accumulate partially glycosylated avenacin A-1, having root epidermis defects and affected saponin cellular distribution (Mylona et al., 2008), illustrating the detrimental effects of pathway intermediates.

Even though saponins are among the largest classes of natural products, their mode of action is not really understood—this may partly be attributed to the often complex mixtures of saponins in plants. Saponins have been shown to cause increased insect mortality, lower food intake, weight reduction, and developmental problems among others (Agerbirk et al., 2003; Christensen et al., 2019); and due to their amphiphilic nature, their most studied effect is regarding their membrane permeabilizing properties. Early studies using artificial lipid bilayers have shown that avenacin A-1 induces permeabilization in a cholesterol-dependent manner and requires the presence of an intact glycoside moiety in the C3 position of the triterpenoid scaffold (Armah et al., 1999). The dependence of the permeabilizing activity on the presence of cholesterol in the membrane and the sugar moiety also was shown for the steroidal saponin digitonin and glycoalkaloids. For example, α-chaconine had strong lytic activity of cholesterol-containing lipid vesicles compared to α-solanine, which has the same aglycone structure but different types of glycosides (Nishikawa et al., 1984; Keukens et al., 1995; Keukens et al., 1996).

**Figure 2:**
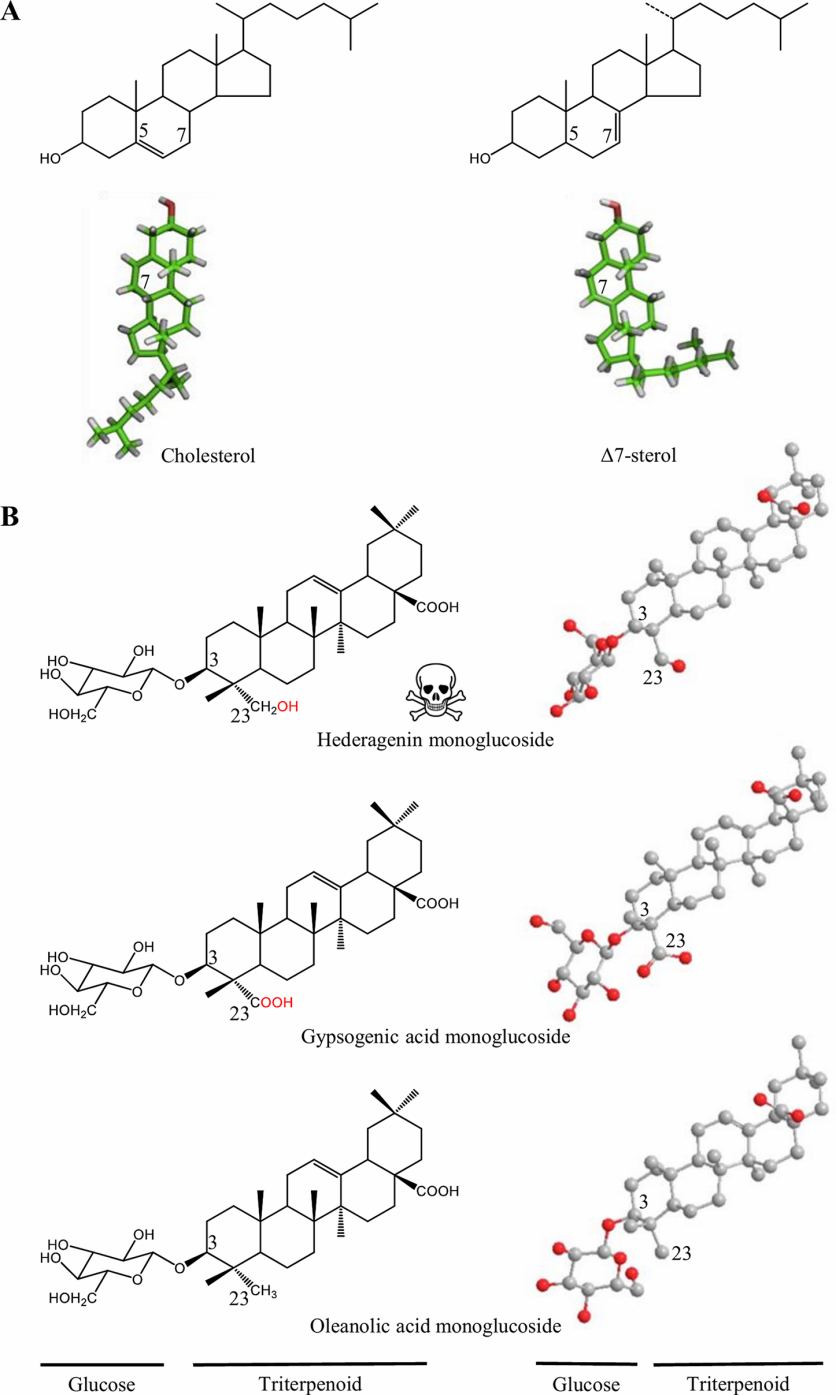
Structural changes may affect the biological activity of triterpenoids. **(A)** Structural formulae (up) and 3D models (down) of cholesterol and Δ7 sterol. In the Δ7 sterol the side chain is bent as compared to cholesterol. In sea cucumber cell membrane systems, cholesterol are replaced by Δ7 sterols to possible modulate the lytic action of saponins as the 3D structure is altered (Claereboudt et al., 2018). **(B)** Structural formulae (left) and 3D models (right) of *B. vulgaris* saponins. In the deterrent hederagenin monoglucoside, the glucose (C3) is twisted in respect to the triterpenoid backbone when a hydroxyl group is added at position C23 (Liu et al., 2019). 3D models are derived from Claereboudt et al. (2018) and Liu et al. (2019), respectively.

Consequently, a prominent role in the avoidance of autotoxicity can be attributed to modifications in the cell membrane composition and further modifications on the saponin structure. Saponins are also present in marine organisms such as sea stars and sea cucumbers (Yendo et al., 2014). Interestingly, recent studies on how sea cucumbers tolerate the toxic saponins they produce, suggest sea cucumbers have replaced cholesterol by other sterols (e.g., Δ7 and Δ9 sterols) in their membranes, presumably in order to modulate the lytic action of its own saponins (Popov, 2003). A detailed analysis of the sterols present in sea cucumbers showed that although cholesterol and Δ7 sterols have essentially the same chemical formula and molecular weight (**Figure 2**), the double bound present in Δ7 sterols has a dramatic effect on its 3D structure, possibly affecting its molecular interaction with sea cucumbers own saponins (Claereboudt et al., 2018). A similar effect on the 3D structure has been speculated to play a role in the biological activity of saponins from *B. vulgaris* (**Figure 2**). The hydroxylation of oleanolic acid at position C23 catalyzed by CYP72A552, leading to the formation of the highly-deterrent hederagenin causes a rotation of the monoglucoside at C3 of *c.* 90°, relative to the plane of the aglycone. When a carboxyl group is introduced instead as in gypsogenic acid, the glucose is situated about the same plane as in oleanolic acid. Gypsogenic and oleanolic acid glycosides are much less toxic to larvae of both *Manduca sexta* and *Plutella xylostella* than they are compared to hederagenin monoglucoside (Liu et al., 2019). Accordingly, the rotation of the sugar moiety in hederagenin glycosides may be responsible for changes on its physiochemical properties with membrane sterols, and this could lead to alter its biological role.

## Concluding Remarks

Increasing characterization of biosynthetic pathways for plant specialized metabolites is revealing that convergent evolution is surprisingly common (Pichersky and Lewinsohn, 2011). Nevertheless, we lack a biological understanding of how identical classes of specialized metabolites evolved recurrently across lineages and even across Kingdoms of Life. Biochemical analysis of the enzymes combined with molecular phylogenetic analysis have until now given detailed insights of how the enzymes for these compounds might have evolved. Further studies are needed to elucidate which biological pressures drive the recurrent emergence of the same or similar specialized metabolites across lineages of plants.

Genome analysis have revealed that some triterpenoid pathways are unlinked while others are organized in operon like clusters. The driving forces behind the genome organization of triterpenoid pathways is currently not understood and difficult to address experimentally. However, as more plant genomes become readily available this key question in evolution of triterpenoid diversity might become addressable.

## Author Contributions

All the authors conceived and wrote the manuscript.

## Funding

PC work was supported by the European Union’s Horizon 2020 research and innovation programme under the Marie Sklodowska-Curie grant agreement No. 752437. SB and AA were supported by grants from the Independent Research Fund Denmark grant No. 7017-00275B and Novo Nordisk Foundation grant No. NNF17OC0027646.

## Conflict of Interest

The authors declare that the research was conducted in the absence of any commercial or financial relationships that could be construed as a potential conflict of interest.
